# Severe bradycardia in a teenager as the initial manifestation for Guillain Barré syndrome: a case report

**DOI:** 10.1186/s12887-022-03165-w

**Published:** 2022-03-22

**Authors:** C. Bonilla, M. I. Alvarez-Olmos, C. Uribe, J. Fernández-Sarmiento

**Affiliations:** 1 Pediatric Cardiovascular Intensive Care Unit, Fundación Cardioinfantil IC University Hospital. Pediatric Intensive Care Unit, Fundación Santa Fe de Bogotá University Hospital, Bogotá, Colombia; 2 Section of Pediatric Infectious Diseases, Fundación Cardioinfantil IC University Hospital. Pediatric Infectious Diseases Fellowship Program, Universidad El Bosque, Bogotá, Colombia; 3 Medical doctor Universidad de los Andes, Pediatric resident Fundación Santa Fe de Bogotá University Hospital, Bogotá, Colombia; 4 Pediatric Intensive Care Unit, Fundación Cardioinfantil IC University Hospital. Pediatric Intensive Care Fellowship Program, La Sabana University, Bogotá, Colombia

**Keywords:** Flaccid paralysis, Variant, Autonomic dysfunction, Pediatric, Plasmapheresis

## Abstract

**Background:**

Guillain-Barré syndrome is the most common cause of flaccid paralysis, with multiple known clinical variants. Autonomic dysfunction, although frequently reported in the clinical course, is often overlooked in the pediatric population and is usually not the initial presenting symptom in this age group

**Case presentation:**

We present the case of a previously healthy 17-year-old who arrived at the Emergency Department complaining of gastrointestinal symptoms associated with lipothymia. An initial electrocardiogram (ECG) showed sustained sinus bradycardia subsequently associated with arterial hypertension. Structural and inflammatory cardiac pathology were ruled out, as well as auriculoventricular conduction block and posterior reversible encephalopathy syndrome. On the ninth day after initial symptoms, the patient presented sensory and motor nerve disturbances with the cerebrospinal fluid analysis showing a clear albumin-cytologic dissociation, consistent with an atypical presentation of GBS with autonomic dysfunction. Immunoglobulin therapy was administered, developing subsequent aseptic meningitis, that required discontinuation of previous therapy and treatment with plasmapheresis. Clinical improvement was achieved with full motor function recovery.

**Conclusion:**

This case illustrates a Guillain-Barré syndrome variant in which autonomic dysfunction preceded neurologic deficit, a finding uncommon in children, emphasizing this as an important differential diagnosis for severe bradycardia in pediatric patients.

## Background

Autonomic dysfunction, which can occur in 2/3 cases of Guillain-Barré syndrome as an acute presentation before the development of sensory or motor symptoms, has been associated with worse outcomes [1]. Although this presentation is infrequent and difficult to diagnose, this phenotypic variant, with restricted autonomic manifestations, constitutes an acute inflammatory neuropathic event caused by immune-mediated mechanisms that may be successfully treated with plasmapheresis or intravenous immunoglobulin [1, 2].

## Case presentation

**Fig. 1:**
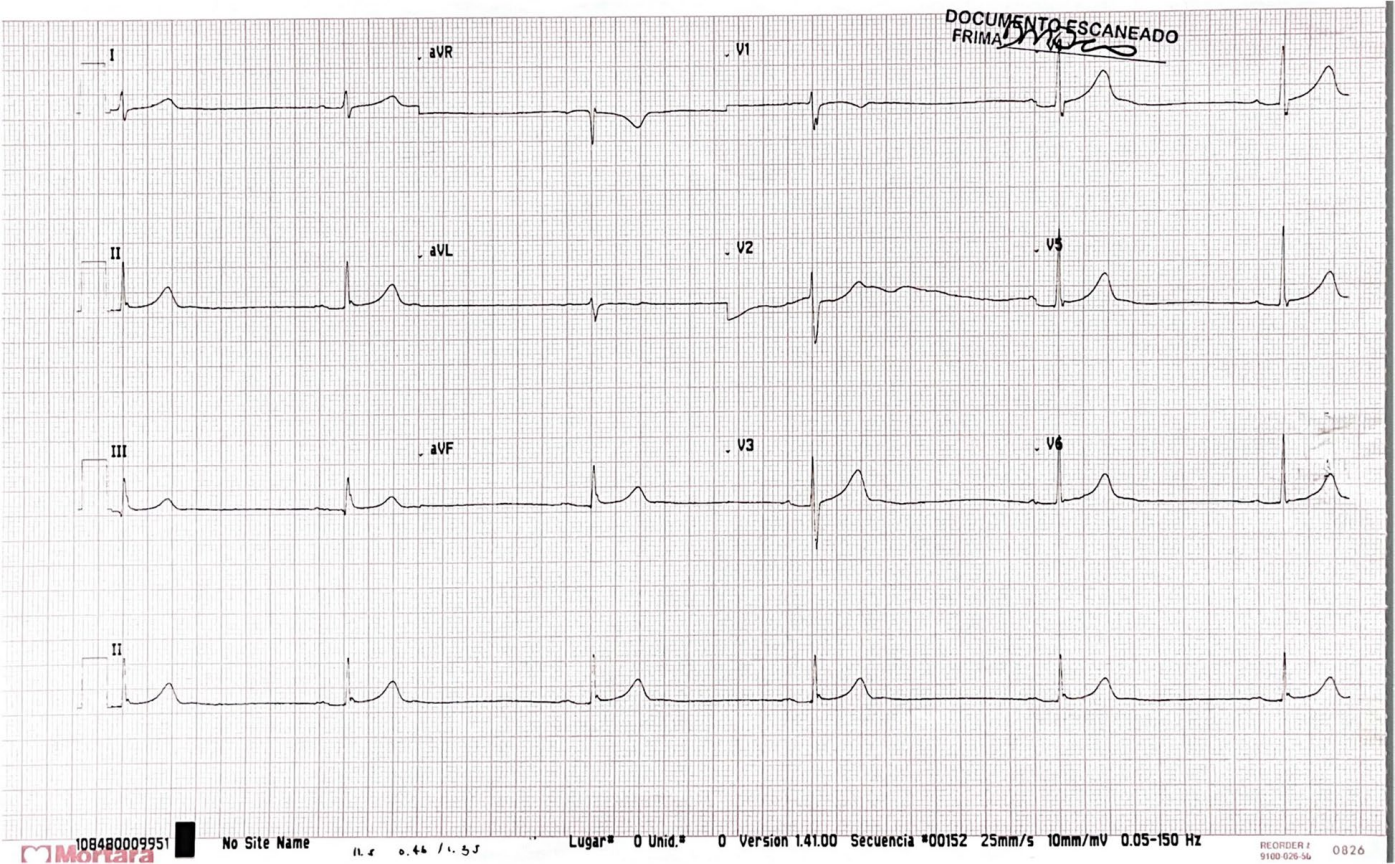
Patient electrocardiogram reading RS, 32 beats per minute, PR 190 ms, QRS 100 ms, cQT 420 mms, 90°

**Fig. 2:**
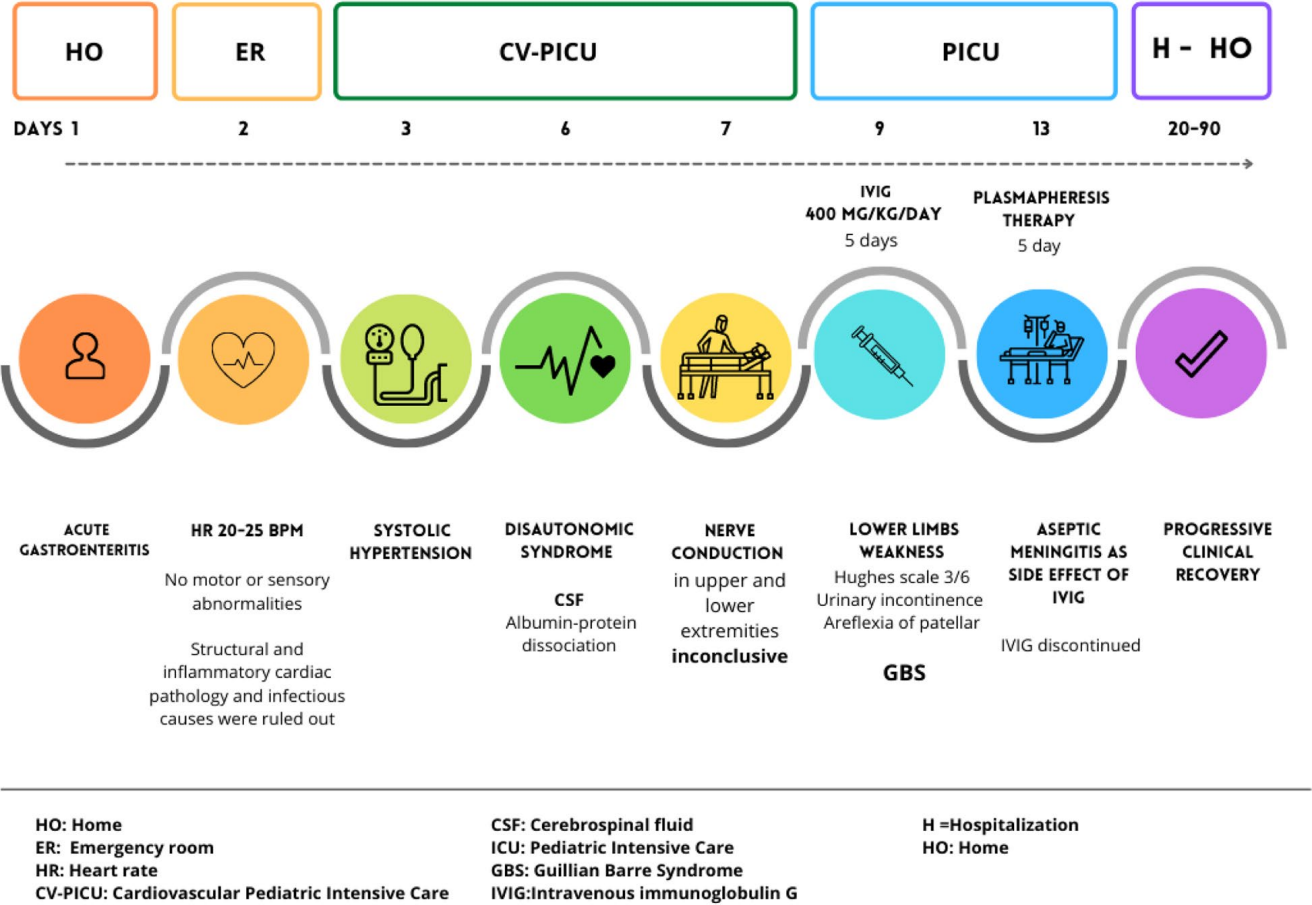
Timeline with relevant data from the episode of care

A previously healthy, 17-year-old male adolescent arrived at the Emergency Room Department on mid-February 2020 complaining of acute-onset of left flank abdominal pain, intensity 5/10, associated with diarrhea and lipothymia. Initial evaluation revealed a heart rate of 20-27 beats per minute (Figs. 1 and 2), requiring immediate transfer to the Pediatric Cardiovascular Intensive Care Unit for further evaluation and monitoring.

Due to the abdominal pain and diarrhea, an infectious etiology was considered and, following blood, urine and stool cultures, treatment with ceftriaxone was initiated. Laboratory evaluation revealed complete cell blood count, glucose, venous gases, renal function, hepatic function, and serum electrolytes (sodium 139 mEq/L, chloride 110 mEq/L, potassium 4.1mEq/L, calcium 8.9 mg/dL, phosphorus 5 mg/dL, magnesium 1,9 mg/dL) all within normal limits; toxicology screen was negative. Contrasted abdominal CT showed mild concentric wall thickening of descending and sigmoid colon, and inflammatory periportal and perivascular reaction in the soft tissue. Mild leukopenia was observed (WBC count of 4860/mm^3^), with normal erythrocyte and platelet counts, and a negative C reactive protein (0.01 mg/dL). Early improvement of acute gastroenteritis was observed, and antibiotic treatment was discontinued soon after blood and stool cultures results were negative.

Structural cardiac pathology was ruled out by a normal transthoracic echocardiogram. Holter monitoring showed sinusal bradycardia without bundle branch block or other conduction abnormalities, suggesting possible autonomic dysfunction without pacemaker indication (Fig. 1). Cardiac enzymes (troponin, CPK, CPK-MB) ruled out an acute inflammatory process, and alfa-1-adrenergic treatment with Midodrine 2.5 mg every 8 hours with the objective of generating venous vasoconstriction was initiated.

Three days following his admission, he became hypertensive, with systolic arterial pressure readings above 170 mmHg and diastolic levels of 104 mmHg. Renal Doppler ultrasound and ophthalmologic evaluation excluded chronic causes of hypertension. Thyroid and renal function tests, aldosterone, renin, and serum/urine catecholamines were normal. Six days after admission to the Intensive Care Unit, a neuropediatric consult was ordered to evaluate dysautonomic symptoms without sensory, motor or any other pupillary dysfunction present. Diarrheal stools completely resolved during the first day of admission.

Considering persistent vagal-adrenergic autonomic compromise with autonomic dysfunction, associated with initial presenting symptoms suggestive of infective gastroenteritis, dysautonomia variant of Guillain-Barré syndrome was considered. A cerebrospinal fluid analysis showed leucocytes (0.0/mm^3^), erythrocytes (0.0/mm^3^), glucose (52 mg/dL), and proteins (594.2 mg/dL) with clear albumin-cytologic dissociation.

Initial nerve conduction studies with F wave and H reflex evaluation in upper and lower extremities demonstrated normal motor unit morphology, without signs of acute or chronic denervation; discrete decrease in proximal and distal muscle pattern recruitment in all four limbs was subsequently confirmed, with interference pattern of 90% (non-conclusive for acute denervation injury). A gastrointestinal Multiplex Polymerase Chain Reaction (PCR) testing by Film-Array® ruled out *Campylobacter* spp., *Yersinia* spp., enterovirus, and other enteric pathogens. Cerebral magnetic resonance was normal, with no evidence of parietal or occipital lesions consistent with posterior reversible encephalopathy syndrome in the context of dysautonomia and severe hypertension. Simple and contrasted sella turcica and cervical, thoracic, and lumbosacral imaging were all normal.

On the ninth day of admission, progressive weakness was observed and associated with extension to the hips resulting in difficulty walking, one episode of urinary incontinence, patellar areflexia, and strength 2+/5 bilaterally in lower limbs. Neither upper limbs, cranial nerves, deglutition nor respiratory muscles were compromised. Hughes scale 3/6 for Guillain-Barré was confirmed.

Intravenous human immunoglobulin G (IVIG) therapy (400 mg/Kg/day) was ordered for 5 days; however, on the third day of treatment, the patient developed a severe headache requiring management with intravenous paracetamol, pregabalin and oxycodone. A simple cranial CT scan was normal, without evidence of acute hemorrhage or edema. Cerebral MRI showed increased leptomeningeal enhancement, particularly in the posterior fossa, compatible with uncomplicated meningitis. A second lumbar puncture was performed, which reported aseptic cerebrospinal fluid consistent with an acute inflammatory process, with a predominance of lymphocytes, hypoglycorrhachia and increased protein concentration. Aseptic meningitis secondary to immunoglobulin therapy was diagnosed, requiring switching to plasmapheresis for Guillain-Barré, and symptom relief was achieved after five sessions. Prior to discharge, patient was able to get independent march. Four months after discharge, he was able to walk and climb stairs without assistance. Complete clinical recovery was observed after 6 months.

## Discussion and conclusions

Previous publications have reported that up to 67% of patients with Guillain-Barré Syndrome (GBS) may present with autonomic disfunction associated with cardiovascular complications such as bradycardia, auriculoventricular block, sinus arrest, sustained sinus tachycardia, and ventricular arrythmias [1, 2]. Moreover, blood pressure fluctuations, vasomotor dysfunction and intestinal motility dysregulation has also been reported [2]. Although autonomic dysfunction was first described in 1892 [3], in recent decades the need for early recognition and treatment has been emphasized to improve mortality rates, which can reach 7% [4, 5].

*Campylobacter jejuni* is one of the most frequently agents capable of triggering GBS and the infections usually precedes axonal GBS disease in up to 33% of patients. Furthermore, this infection can result in major damage in patients with GBS compared with other infections [6].

Our patient developed typical presentation of GBS nine days after the initial symptoms of gastroenteritis with negative stool culture for *C. jejuni*. Although serology testing may be a potential option for diagnosing this infectious agent, this tool is not available in our center. A multiplex PCR (Film-Array®) was performed in stool to identify an infectious cause of gastroenteritis episode without success. There is not a clear etiologic explanation for his initial enteritis and dysautonomic disfunction. However, the diarrheal episode resolved fast, and constipation rapidly began when the dysautonomia became present. At the time of our patient ER consultation on mid of February 2020, SARS-CoV-2 testing was not available since the epidemic was declared until March 2020 and the first local case was reported 3 weeks after his hospital admission.

GBS patients may develop blood pressure fluctuations, such as arterial hypertension (27% of patients), and transitory hypotension or sustained hypotension (3% of cases). Drastic fluctuations between severe hypertension and hypotension may cause cardiovascular collapse and sudden death [4]. These fluctuations are related with increased afferent sympathetic tone, transitory serum catecholamine levels, baroreceptor dysregulation, preganglionic sympathetic axonal demyelination, and/or postganglionic axonal degeneration, producing altered feedback or inappropriate ectopic electrical discharges [7].

To date, the relationship between autonomic nervous system disfunction (blood pressure fluctuations and vasomotor abnormalities) and the degree of severity in GBS patients is unclear. There is not a currently sensitive marker to predict early identification of arrythmia in those who will likely develop clinic of GBS [8]. Currently known causes of arrhythmias include [2]: 1) autonomic afferent demyelination from the heart; 2) respiratory failure; 3) Brainstem central vagal control (nuclear lesions); 4) independent risk factors for autonomic cardiac innervation, such as direct interstitial infiltrate, with or without myocardial necrosis

Pupillary dysfunction can also be observed on physical examination in up to 14 % of patients with GBS [1], although diagnosis may improve if automatized pupillometry is routinely performed, offering a more objective, accurate and quantitative evaluation of pupillary function (including size and constriction/dilation velocity) [9].

Paralytic ileus has been described in up to 15% of GBS patients secondary to lack of intrinsic neuronal control and extrinsic gastrointestinal pathways, with compromised parasympathetic and sympathetic autonomic nerve fibers [10]. Gastroparesis, delayed gastric emptying, diarrhea as may have occurred in our patient, and/or fecal incontinence are not uncommon findings. It is unclear whether these findings are all secondary to dysautonomia, immobility, or secondary effects of medication such as opioids. Moreover, altered peptide secretion, decreased mesenteric blood flow and gastrointestinal neural pathways have all been hypothesized to play a role.

Identifying autonomic neuropathy as a dysautonomic variant of GBS in the early stages of disease is of upmost importance. Although many different autonomic tests have been developed (e.g., eyeball pressure test, 24-h heart rate power spectrum, head-up tilt test, hand grip testing, cold pressor test, atropine test, catecholamine testing, microneurographic studies and sudomotor testing), studies related to reproducibility, interobserver variability, and predictive value in patients with GBS are lacking [11].

Additionally, studies evaluating blood and urine neurotransmitter levels as substitute markers for the autonomic function in GBS patients, as opposed to vanilmandelic acid and catecholamines, are limited [12]. Moreover, sensory and motor nervous conduction studies through electromyography are not always accurate in GBS variants, as electrophysiologic abnormalities may be mild or unspecific in early disease stages [13].

GBS as an autoimmune polyradiculoneuropathy shows adequate response to early immunotherapy with plasmapheresis or IVIG, improving autonomic symptoms. Although superiority or non-inferiority trials are inconclusive, IVIG is usually preferred as first-line treatment due to its relative simplicity of administration compared to plasmapheresis, as well as greater availability. Recommended dose of 1 g/Kg/day for 2 days or 400 mg/Kg/day for 5 days of IVIG may suppress the inflammatory response [1, 2, 13]. Additional analysis is required to establish which immunotherapy modality is useful and if plasmapheresis is in fact better than IVIG in GBS with autonomic dysfunction, considering possible autonomic dysfunction symptom presentation versus adverse effects from treatment including hypotension or hypertension due to electrolyte imbalance generated by plasmapheresis. However, patients with autonomic dysfunction require aggressive treatment, either with plasmapheresis or IVIG. Our patient required switching treatment modality due to aseptic meningitis, which can be seen as an infrequent adverse event of IVIG administration, with excellent clinical improvement following the use of plasmapheresis.

Mild to severe motor disability in GBS may appear in 30% of patients who develop vagal hyperactivity [2]. Nonetheless, little is known about GBS in pediatric patients regarding this presentation and its prognosis.

Published literature about autonomic dysfunction and progression to GBS indicates that this may be a transitory phenomenon, with resolution in months [1, 2]. Although an increase in mortality has been reported in patients with autonomic dysfunction [1], prospective and multicentric studies looking at the relation between autonomic dysfunction presentation and mortality risk are lacking.

This case illustrates the presence of a GBS variant where autonomic dysfunction, with profound bradycardia and blood pressure fluctuations, preceded the expected neurologic deficit. Early identification of these signs assured early diagnostic testing and opportune treatment options. Although not all testing evaluations are performed as first line options, close monitoring, and a multidisciplinary approach in patients with GBS with autonomic dysfunction variant is necessary for appropriate management and diminishing morbimortality [14].

## Data Availability

Not applicable. All resources cited throughout the text are referenced in the Bibliography section.

## Abbreviations

WBC: White blood cells
CT: Computed tomography
EKG: Electrocardiogram
CPK: Creatine phosphokinase
CPK-MB: Creatine phosphokinase isoenzyme MB
GBS: Guillain-Barré syndrome
IVIG: Intravenous immunoglobulin
